# Effect of Black Garlic on Microbiological Properties, Lipid Oxidation, Residual Nitrite, Nitrosamine Formation and Sensory Characteristics in a Semi-Dry Fermented Sausage

**DOI:** 10.3390/foods12071545

**Published:** 2023-04-06

**Authors:** Begüm Akansel, Zeynep Feyza Yılmaz Oral, Selen Sallan, Güzin Kaban, Mükerrem Kaya

**Affiliations:** 1 Department of Food Engineering, Faculty of Agriculture, Atatürk University, TR-25240 Erzurum, Türkiye; 2 Department of Food Technology, Vocational School of Technical Sciences, Atatürk University, TR-25240 Erzurum, Türkiye; 3 Departmet of Food Processing, Bandırma Vocational School, Bandırma Onyedi Eylül University, TR-10200 Balıkesir, Türkiye; 4 MK Consulting, Ata Teknokent, TR-25240 Erzurum, Türkiye

**Keywords:** fermented sausage, black garlic, nitrosamine, *N*-nitrosodimethylamine, *N*-nitrosodiethylamine, *N*-nitrosopiperidine

## Abstract

This study was conducted with the aim of determining the effects of different black garlic (BG) levels (1%, 2% and 3%) on quality characteristics of a semi-dry fermented sausage (heat-treated sucuk). In addition, the effect of cooking time (0, 1 or 3 min at 180 °C on a hot plate) on nitrosamine formation was investigated. Fresh garlic (FG, 1%) was evaluated as the control group. BG (2% and 3%) caused a reduction in the count of lactic acid bacteria while leading to an increase in pH. FG1% gave the highest number of *Micrococcus*/*Staphylococcus*, as well as a_w_ value. The thiobarbituric acid reactive substance (TBARS) value increased with increasing BG levels. FG (1%) showed the highest residual nitrite amount (*p* < 0.05). The scores for color, taste and general acceptability were reduced by the use of BG (*p* < 0.05). No significant difference was observed between the garlic treatments in terms of *N*-Nitrosodimethylamine (NDMA) and *N*-Nitrosodiethylamine (NDEA) when no additional cooking was applied. Cooking time was determined to have no significant effect on NDMA in 3% BG. The use of BG caused an increase in *N*-Nitrosopiperidine (NPIP) (*p* < 0.05). As for PCA, a closer correlation between NPIP and the groups containing BG was observed, while there was a strong correlation between NDMA and the FG group cooked for 3 min. The use of BG caused an increase in NPIP, but affected NDMA and NDEA depending on the cooking time.

## 1. Introduction

Fermented sausages include dry or semi-dry products such as salami, chorizo, hard salami, pepperoni, thuringer, summer sausage, Rohwurst, cervelat, sucuk and heat-treated sucuk. Fermented sausages are considered safe products due to hurdle effects such as pH, a_w_, nitrite and redox potential. The a_w_ value in dry fermented sausages is below 0.90, while it varies between 0.90 and 0.95 in semi-dry fermented sausages [1]. The ingoing nitrite level is an important factor for product safety. Nitrite is mostly used in rapid-ripened products, while nitrate is preferred in long-ripened products [2]. However, nitrate must be converted to nitrite in order to observe the expected effects of this curing agent in processes using nitrate [3]. Nitrite, which has multifunctional properties (antioxidant, antimicrobial, development of characteristic flavor and formation of color properties), is also involved in the formation of nitrosamines [4,5].

Nitrosamines are compounds with carcinogenic, mutagenic and teratogenic properties [4]. *N*-Nitrosodimethylamine (NDMA) and *N*-Nitrosodiethylamine (NDEA) are defined by the International Agency for Research on Cancer (IARC) as probably human carcinogens (Group 2A). *N*-Nitrosodibutylamine (NDBA), *N*-Nitrozopiperidine (NPIP), *N*-Nitrozopyrrolidine (NPYR) and *N*-Nitrosomorpholine (NMOR) are among the possibly (Group 2B) human carcinogenic compounds [6]. Among these compounds, NDMA, NPIP and NPYR are commonly found *N*-nitroso compounds in meat products [7]. The nitrosamine content of semi-dry and dry fermented sausages such as salami, raw sausage, cervelat, pepperoni, chorizo, sucuk and heat-treated sucuk can be below the detection limit or reach very high levels [7,8,9,10,11,12,13]. Many studies have been conducted on the use of inhibitors such as ascorbic acid and plant polyphenols [14,15,16], reducing ingoing nitrite level [17] and the use of lactic acid bacteria [18,19,20], with the aim of reducing the amount of nitrosamine in meat products. The effect of garlic, which is rich in sulfur compounds, on the formation of nitrosamines has also been studied, and it was found that it can reduce nitrosamine content due to allyl sulfide compounds [21,22]. Allicin (diallyl thiosulfate) is biologically the most active sulfur compound in garlic. Another chemical group that characterizes garlic are phenolic compounds [23,24]. The amount of these compounds is higher in black garlic [24,25,26]. Black garlic is a processed product obtained by long (several weeks) fermentation of fresh garlic bulbs (*Allium sativum* L.) at high temperature (60–90 °C) and relative humidity (50–95%) without additional heat treatment or additives [26].

Garlic is included in the formulation of many dry and semi-dry fermented sausage varieties [2,27,28,29]. However, due to black garlic’s functional potential, its use in many foods, including sausages, is growing in popularity [30]. Garlic is also included in the formulation of heat-treated sucuk, a semi-dry fermented sausage type widely produced in Türkiye, and is added to the batter at 1% or more. The main processing steps of this product are fermentation (initial fermentation temperature: 22–24 °C), heat treatment (core temperature of 60–68 °C) and drying (moisture: protein < 3.6) [31,32]. Due to consumption habits, heat-treated sucuk is also consumed by cooking. A previous study reported that the cooking process resulted in significant increases in the nitrosamine content of heat-treated sucuk, depending on the degree of cooking applied [16]. Therefore, alternative applications to prevent nitrosamine formation in heat-treated sucuk are very important. Black garlic (BG) has an enhanced antioxidant capacity compared with fresh garlic (FG), and it has many beneficial health effects due to its functional compounds, such as its antioxidant, antidiabetic and anti-inflammatory activities [25,26]. Based on this trait, it was hypothesized that BG could prevent nitrosamine formation in heat-treated sucuk. The effects of BG on nitrosamine formation in fermented sausages have not been studied yet. In addition, the effect of BG on the quality properties of fermented sausages was not examined. In this study, the aim was to determine the effects of using FG (1%) and BG at different levels (1%, 2% and 3%) on the microbiological properties, lipid oxidation, residual nitrite and sensory characteristics of heat-treated sucuk and the formation of nitrosamines.

## 2. Materials and Methods

### 2.1. Material

Meat and fat from the round and chuck parts of three beef carcasses from a local butcher were used. After removing excess fat, beef meat was cut into small pieces and stored at −18 °C until production. Beef fat was also chopped and stored at −18 °C until production.

Taşköprü garlic was used as fresh garlic (FG). According to the Commission Implementing Regulation (EU) 2021/615, the designation of origin of Taşköprü garlic is protected and given the Protected Geographical Indication with the specific name “Taşköprü Sarımsağı”. It was obtained from the commercial company (Orhan Reis Tarım Ürünleri A.Ş., Taşköprü, Kastamonu, Türkiye). Black garlic (BG) was also obtained from the same company. It has been stated by the company that BG is obtained by the fermentation of fresh Taşköprü garlic under controlled conditions. The pH and a_w_ values were determined as 6.33 and 0.973 for FG and 4.48 and 0.907 for BG, respectively.

Autochthonous *Latilactobacillus sakei* S15 and *Staphylococcus xylosus* GM97 strains [33,34] were added to the batters as starter cultures at 10⁷ cfu/g and 10⁶ cfu/g, respectively.

### 2.2. Heat-Treated Sucuk Production

Heat-treated sucuk was produced with 80% lean beef meat and 20% beef fat. The ingredients of the heat-treated sucuk formulation were as follows: 20 g/kg of NaCl, 4 g/kg of sucrose, 7 g/kg of red pepper, 5 g/kg of black pepper, 9 g/kg of cumin, 2.5 g/kg of allspice and 0.15 g/kg of sodium nitrite. The above-mentioned strains in production were included in the formulation. Four different batches of heat-treated sucuk were manufactured: heat-treated sucuk with 1% FG (control batch), heat-treated sucuk with 1% BG, heat-treated sucuk with 2% BG and heat-treated sucuk with 3% BG. For each of the four treatments, three independent batches of sausages were prepared; thus, a total of twelve batches of sausage were made. Batches were prepared using a laboratory-type cutter (MADO Typ MTK 662, Dornhan/Schwarzwald, Germany) to a final particle size of almost 3 mm. Batches of 200 ± 10 g were stuffed in collagen casings 38 mm in diameter (Naturin GmbH & Co., Weinheim, Germany) with a filling machine (MADO Typ MTK 591, Dornhan/Schwarzwald, Germany).

Heat-treated sucuk groups were fermented in an automatic climate unit (Reich, Thermoprozestechnik GmbH, Schechingen, Germany) at 24 ± 1 °C at 90 ± 2% relative humidity for 24 h. Following the fermentation, they were subjected to heat treatment in a steam cooking chamber (Mauting, Valtice, Czechia) to reach an internal temperature of 64 °C. After heat treatment, the treatment groups were dried in an automatic climate unit (Reich, Thermoprozestechnik GmbH, Schechingen, Germany) for 48 h at 18 ± 1 °C and 84% ± 2 relative humidity.

### 2.3. Cooking Procedure for Heat-Treated Sucuk

The heat-treated sucuk samples were sliced to 0.5 mm thick at the end of the production. Cooking was performed on a hot plate. The surface temperature of the plate was raised to 180 °C before cooking and its temperature was controlled by measurement with a digital thermocouple (Testo 926, Testo, Titisee-Neustadt, Germany). In total, 1 min (0.5 min per surface) and 3 min (1.5 min per surface) were applied as cooking times. Samples without additional cooking were considered as control (0 min). Each sample was homogenized, placed in a glass jar and then frozen at −20 °C until analysis.

### 2.4. Analysis

The final products obtained after drying were subjected to microbiological, pH, a_w_, TBARS, residual nitrite and sensory analyses. Nitrosamine analysis was performed to both uncooked and cooked samples. All analyses were replicated twice.

#### 2.4.1. Microbiological Analysis

To determine the number of lactic acid bacteria of the samples, De Man Rogosa Sharpe Agar (MRS, Merck, Darmstadt, Germany) was used. MRS plates were incubated at 30 °C under anaerobic conditions (Anaerocoult A, Merck). The number of *Micrococcus/Staphylococcus* was determined using Mannitol Salt Phenol Red Agar (MSA, Merck) after aerobic incubation at 30 °C for 2 days. The number of Enterobacteriaceae was determined using Violet Red Bile Dextrose Agar (VRBD, Merck). Petri plates were incubated at 30 °C for 2 days under anaerobic conditions (Anaerocoult A, Merck). The results were converted to the logarithm of the number of colony forming units (log cfu/g) [1].

#### 2.4.2. Physicochemical Analysis

To determine a_w,_ a water activity device (Novasina AG CH-8853, Lachen, Switzerland) was used and calibrated at 25 °C with six different salt solutions before use. a_w_ values were determined at 25 °C [35].

For pH, 10 g of analysis sample was mixed with distilled water (100 mL) and homogenized (1 min) with ultra turrax (IKA Werk T25, Staufen im Breisgau, Germany). The pH value of the homogenate was measured with a pH meter (Mettler Toledo, Greifensee, Switzerland). Before using the pH meter, it was calibrated with appropriate buffer solutions (pH 4.0 and 7.0) [35].

The thiobarbituric acid reactive substances (TBARS) value was determined according to the method given by Lemon [36]. The result was given as mg MDA/kg.

To determine residual nitrite, 10 g sample was mixed with 50 mL ultrapure water (50–60 °C) and transferred to a 200 mL flask. Then, 50 mL of acetonitrile was added and, after stirring for a further 15 min, the volume was made up to 200 mL with ultrapure water. Samples filtered with nitrite-free/nitrate-free filter paper (MN 640 de, Macherey-Nagel) were passed through a 0.45 µm filter and taken into vials. The residual nitrite content was determined using high-performance liquid chromatography (HPLC) coupled with a diode array detector (DAD) (Agilent Technology, Santa Clara, CA, USA). The flow rate in the system was 2 mL/min. Detecting UV wavelength was 220 nm. The injection volume was taken to be 100 µL. Results are reported in mg/kg based on the calibration carried out with the nitrite standard [37].

For the nitrosamine analysis, the 10 g sample was placed in centrifuge tubes and homogenized after the addition of 0.1 M NaOH solution. Then, methanol was added and the sample was centrifuged at 10.000 rpm for 10 min at 4 °C. Thereafter, the sample was filtered with glass microfiber (70 mm diameter, Whatman GF Healthcare Life Sciences UK) and transferred to the ChemElut column (Agilent ChemElut, 20 mL, unbuffered, Santa Clara, CA, USA) by adding 20% NaCl solution to the extract. Then, dichloromethane was added and the mixture was concentrated to 1 mL with a Kuderna Danish apparatus. The concentrate was evaporated under nitrogen at 40 °C and GC/MS was used to detect nitrosamines. Helium was used as the carrier gas. DB-5MS was used as column and operated in SIM mode. After the oven temperature was kept at 50 °C for 2 min, it was increased to 100 °C at a rate of 3 °C/min and held at this temperature for 5 min. The temperature was then increased to 250 °C at a rate of 20 °C/min. Nitrosamine mix (EPA 521 nitrosamine Mix, Supelco, Bellefonte, PA, USA) was used for identification and nitrosamine amounts (*N*-Nitrosodimethylamine (NDMA), *N*-Nitrosodiethylamine (NDEA), *N*-Nitrosodibutylamine (NDBA), *N*-Nitrozopiperidine (NPIP), *N*-Nitrozopyrrolidine (NPYR), *N*-Nitrosomethylethylamine (NMEA) and *N*-Nitrosodipropylamine (NDPA)) were determined at μg/kg level [15].

#### 2.4.3. Sensory Analysis

The sensory evaluation of heat-treated sucuk was conducted by 20 panelists. The selection of panelists was based on the experience of pre-sensory testing, and they were informed about the sensory analysis process, i.e., they were given information about the score card and the evaluation procedures.

Each individual panelist was asked to evaluate the sensory attributes of sausage samples, including color, texture, odor, taste and overall acceptability. A nine-point hedonic scale (one = strongly dislike, nine = strongly like) was used to score the samples. Evaluations were carried out under natural light in a quiet laboratory at room temperature (22 ± 2 °C) [35].

#### 2.4.4. Statistical Analysis

The experimental design was performed according to a randomized complete block design (three blocks). Microbiological analysis, pH, a_w_, TBARS, residual nitrite and sensory evaluation data were analyzed using the general linear model (GLM) ANOVA procedure, which contained the fixed effect of garlic treatment (FG 1%; BG 1%, 2% or 3%). For microbiological analysis, pH, a_w_, TBARS, residual nitrite and sensory evaluation, the following mathematical model was used:

Y_ij_ = μ + b_i_ + g_j_ + e_ij_ (1)

Y_ij_ is the response variable; μ is the overall mean; b_i_ is the i^th^ block effect; g_j_ is the fixed effect of garlic treatment; e_ij_ is random error.

Nitrosamine results were also analyzed using the GLM. The model used accounted for the fixed effects of garlic treatment, cooking time (0 min, 1 min or 3 min) and the interaction between garlic treatment and cooking time. The production of heat-treated sucuk was performed in triplicate, and three blocks were evaluated as random effect in the model. The following mathematical model was used for the nitrosamine analysis:

Y_ijk_ = μ + b_i_ + g_j_ + c_k_ + (gc)_jk_ + e_ijk_ (2)

Y_ijk_ is the response variable; μ is the overall mean; b_i_ is the ith block effect; g_j_ is the fixed effect of garlic treatment; c_k_ is the kth effect of cooking time; (gc)_jk_ is the jkth interaction effect of garlic treatment and cooking time; e_ijk_ is random error.

When all factors and interactions were significant, Duncan’s multiple range tests were performed at *p* < 0.05 level to compare differences between the means. SPSS version 24 statistical software (SPSS Inc., Chicago, IL, USA) was used for all statistical analyses. Principal component analysis (PCA) was performed to evaluate the relationships between heat-treated sucuk groups with different garlic content (FG-1%, BG-1%, BG-2% and BG-3%), cooking times (0, 1 or 3 min) and nitrosamines detected. The Unscrambler software (CAMO software version 10.1, Norway) was used for PCA analysis. In addition, the relationship between heat-treated sucuk groups without additional heat treatment and physicochemical parameters (pH, TBARS, residual nitrite, NPIP, NDMA and NDEA) was also presented using a heatmap. The heatmap was also applied to the sensory analysis results. A heatmap graph was plotted using heat mapper [38].

## 3. Results and Discussion

### 3.1. Microbiological Counts, pH, a_w_, TBARS and Residual Nitrite

The overall effect of garlic treatment on the microbiological counts, pH, a_w_, TBARS and residual nitrite of heat-treated sucuk are given in Table 1. The use of 2% or 3% level of black garlic (BG) in heat-treated sausage caused a significant reduction in the number of lactic acid bacteria (*p* < 0.05). However, there was no statistically significant difference between 1% BG and 1% level of fresh garlic (FG) (*p* > 0.05). In the production of heat-treated sucuk, FG is an important ingredient in the formulation. According to these results, the use of 1% BG did not cause any significant differences on the behavior of the technologically important lactic acid bacteria (*p* > 0.05). When the BG level was increased to 2% or 3%, a decrease in the number of lactic acid bacteria by about one logarithmic unit was observed (*p* < 0.05). This decline is believed to be due to the partial slowing down of lactic acid bacteria growth due to antimicrobial compounds in BG during the fermentation stage [30]. Fermentation, heat treatment and drying are the main process steps in heat-treated sucuk production. It has been noted in previous studies that lactic acid bacteria can show good growth at the initial fermentation temperature of 22–24 °C. During the heat treatment applied after fermentation, a reduction of up to 5 log units occurs in the number of lactic acid bacteria, depending on the intensity of the heat treatment (usually 60–68 °C). In the drying phase, there are no significant changes in the count [1,31,39]. Since a heat treatment program based on an internal temperature of 64 °C was applied in the present study, the number of lactic acid bacteria ranged from 10^2^ to 10^3^ cfu/g.

Another group of microorganisms that are technologically important in fermented sausages are micrococci and staphylococci. As in lactic acid bacteria, significant reductions occur during heat treatment in these microorganisms [1,40]. In this present study, *Staphyloccus xylosus* GM92 strain was used as a starter culture and added to the batter at a level of 10^6^ cfu/g. In the final product, the number of *Micrococcus/Staphylococcus* varied between 2 and 3 log cfu/g depending on the garlic type and level. As can be seen from Table 1, the group with 1% BG showed about one log unit lower number than the control group (FG 1%). The lowest mean number was determined in the group containing 3% BG, but the mean value of this group was not statistically different from the mean of the 2% BG group (*p* > 0.05). It is estimated that the groups with BG show a lower *Micrococcus/Staphylococcus* count than the group with FG, even at the 1% level, due to the stronger antimicrobial activity of BG [41].

*Enterobacteriaceae* counts in all groups were below the limit of detection (<2 log CFU/g). Acidification during fermentation and heat treatment were thought to have a significant effect on the number of Enterobacteriaceae family members below the detectable limit in all groups. In fact, similar results were obtained in the studies performed on heat-treated sucuk [1,31,39].

No significant changes were observed in the pH value of heat-treated sausage at 1% BG level compared with control (1% FG) (*p* > 0.05). On the other hand, the pH value was higher in the presence of 2% BG or 3% BG compared with the control (Table 1). It was thought that the antimicrobial effect of BG during fermentation slows down the growth and activity of lactic acid bacteria, especially at 2% and 3% levels, and therefore there is a smaller decrease in pH value in the 2% and 3% groups. As a matter of fact, as can be seen from the lactic acid bacteria results, in the presence of 2% or 3% BG, lower lactic acid bacteria counts were observed in the heat-treated sucuk groups subjected to fermentation, heat treatment and drying under the same conditions (Table 1).

The highest mean of a_w_ value in the study was determined in the control group (1% FG). In other groups, the a_w_ value decreased with increasing BG levels (*p* < 0.05). The a_w_ values of FG and BG used in the present study were 0.973 and 0.907, respectively. It was thought that the effect of garlic on the different a_w_ values of heat-treated sucuk groups is more limited. As a matter of fact, meat fermentation is a very complex system, and pH is hereby estimated to be an important factor [2].

TBARS value, which is an indicator of lipid oxidation, was found to be below 1 mg MDA/kg in all heat-treated sucuk groups (except 3% BG). The degree of lipid oxidation is very limited in this type of fermented sausage, as it has a short production period. Similar results were also found by Armutçu et al. [31] and Yılmaz Oral and Kaban [1]. In this present study, the lowest mean of TBARS value was determined in the 1% FG (control) group. As the BG level increased, the TBARS value increased (*p* < 0.05). The highest mean of TBARS value was 1.02 ± 0.005 mg/kg in the 3% BG group. BG, which is richer in antioxidant substances than FG, increased lipid oxidation in heat-treated sucuk. This result indicated that some compounds in BG might have a prooxidant effect. During the fermentation of FG, compounds such as aldehydes, ketones and alcohols are formed as a result of oxidative and hydrolytic changes in the lipids, especially under high temperatures (60–90 °C) and relative humidity (70–90%). These compounds may play a role in a number of chemical reactions, including hydrolysis and oxidation with lipids [42,43].

In the sausage groups, the highest mean value in terms of residual nitrite was determined in the 1% FG (control) group, while lower values were determined in the groups with BG compared with the control. However, the difference between the means of the 2% BG and 3% BG groups was not statistically significant (*p* > 0.05) (Table 1). Low pH plays an important role in the conversion of nitrite to nitric oxide. Low pH resulting from fermentation increases nitrite depletion [7]. In the present study, 2% and 3% BG levels gave lower residual nitrite levels, which is thought to be due to the antioxidant compounds in BG providing reducing conditions for nitric oxide formation in addition to low pH [44]. As a matter of fact, it has been revealed that BG contains more antioxidants than FG [25,45]. On the other hand, the level of residual nitrite in cured meat products is an important factor regarding nitrosamine formation [17].

**Table 1:** The overall effect of garlic treatment on the microbiological counts, pH, a_w_, TBARS and residual nitrite of heat-treated sucuk (mean ± sd).

Treatment	Lactic Acid Bacteria (log cfu/g)	*Micrococcus/Staphylococcus* (log cfu/g)	pH	a_w_	TBARS (mg MDA kg^−1^)	Residual Nitrite (mg kg^−1^)
FG (1%)	3.84 ± 0.27 a	3.64 ± 0.16 a	5.14 ± 0.04 b	0.941 ± 0.002 a	0.58 ± 0.005 d	19.93 ± 3.86 a
BG (1%)	3.59 ± 0.55 a	2.67 ± 0.43 b	5.16 ± 0.04 b	0.938 ± 0.002 b	0.71 ± 0.004 c	18.40 ± 3.46 b
BG (2%)	2.75 ± 0.45 b	2.1 ± 0.15 c	5.20 ± 0.04 a	0.935 ± 0.002 c	0.90 ± 0.002 b	17.20 ± 2.67 bc
BG (3%)	2.62 ± 0.24 b	2.0 ± 0.00 c	5.22 ± 0.04 a	0.934 ± 0.002 c	1.02 ± 0.005 a	16.47 ± 3.86 c
*Significance*	**	**	**	**	**	**

FG: fresh garlic; BG: black garlic; **: *p* < 0.01; sd: standard deviation; a–d: means marked with different letters in the same column are statistically different (*p* < 0.05).

### 3.2. Sensory Characteristics

The results of sensory analyses of heat-treated sucuk samples are shown in Table 2. According to the sensory panel evaluation, the lowest color score was observed in 1% FG. The color score also increased with increasing BG levels, but the difference between 2% BG and 3% BG levels was not statistically significant. The addition of BG instead of FG significantly reduced the taste scores in heat-treated sucuk (*p* < 0.05). No significant changes were determined between all groups for texture. The use of 1% BG showed no significant differences in the odor score compared with 1% FG (*p* > 0.05). Using 2% BG or 3% BG reduced the odor score (*p* < 0.05). However, no significant differences were found between these two groups in terms of odor score (*p* > 0.05). The general acceptability was the highest in the group with 1% FG (*p* < 0.05), and no significant difference was found between the different levels of BG in terms of general acceptability (*p* > 0.05). However, sensory scores were within acceptable limits in all groups. In other words, no value below 7.00 was detected (Table 2).

### 3.3. Volatile Nitrosamines

*N*-Nitrosodimethylamine (NDMA), *N*-Nitrosodiethylamine (NDEA) and *N*-Nitrozopiperidine (NPIP) were determined in heat-treated sucuk samples; their limit of detection (LOD) values were 0.32 μg/L, 0.44 μg/L and 0.32 μg/L, respectively. *N*-Nitrozopyrrolidine (NPYR), *N*-Nitrosomethylethylamine (NMEA), *N*-Nitrosodipropylamine (NDPA) and *N*-Nitrosodibutylamine (NDBA) were not found in any of the samples. The correlation coefficient (R^2^) was 0.9999. Recovery (%) was detected as 101.00–104.37 for NDMA, 94.00–101.36 for NDEA and 99.73–100.83 for NPIP. The overall effects of garlic treatment and cooking time on nitrosamine contents of heat-treated sucuk are given in Table 3.

#### 3.3.1. *N*-Nitrosodimethylamine (NDMA)

**Figure 1:**
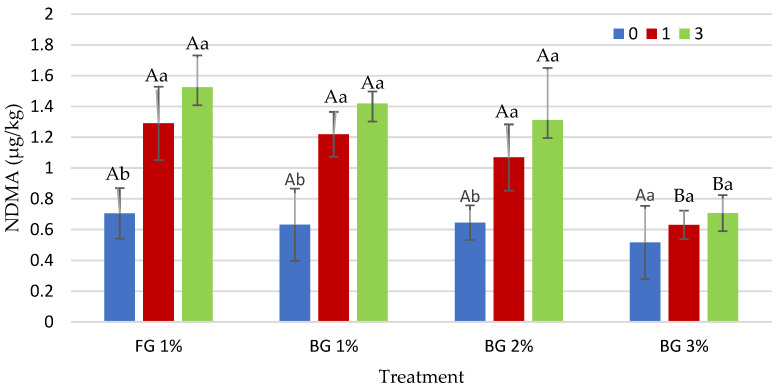
Effect of interaction of garlic treatment × cooking time on *N*-Nitrosodimethylamine (NDMA) content of heat-treated sucuk (A, B: Different capital letters indicate significant differences between groups with garlic for cooking time. a, b: Different small letters indicate significant differences between cooking time for garlic treatment).

The lowest average NDMA value was determined in the group with 3% BG (Table 3). On the other hand, cooking temperature and cooking time are important factors in the increase in NDMA formation. In many previous studies, it has been reported that nitrosamine content increases with increasing cooking intensity [16,17]. In our study, the mean NDMA increased as the cooking time increased (Table 3). However, the garlic treatment × cooking time interaction significantly affected NDMA (*p* < 0.01) (Table 3). No significant differences were detected among the groups (FG 1%, BG 1%, BG 2% and BG 3%) in terms of NDMA in heat-treated sucuk samples when no additional cooking was performed. In the 1 min cooking process, lower NDMA content was observed in 3% BG than the other groups. A similar result was also obtained in the cooking time of 3 min (Figure 1). The NDMA amount increased as the cooking time increased in FG 1%, BG 1% and BG 2%; however, no significant differences were obtained in terms of NDMA content between 1 and 3 min in all three treatments. On the other hand, there were no significant effects of cooking time in 3% BG (Figure 1). As can be seen from the residual nitrite results, lower residual nitrite amounts were obtained with increasing BG levels (Table 3) and, therefore, it was thought that the lower NDMA value at 3% BG was related to the residual nitrite level. Indeed, residual nitrite is believed to be an important factor in nitrosamine formation [46]. In addition, BG rich in phenolic compounds and sulfurous compounds is thought to show its antioxidant effect in 3% BG group depending on the concentration [21,26,45]. Changes in physicochemical properties during the production of BG significantly increase the bioactivity of BG [26]. Under acidic conditions, phenolic compounds react with nitrite more rapidly than many amino compounds. 1,2- and 1,4-dihydroxyphenols, 1,2,3-trihydroxyphenol and many naturally occurring polyphenols inhibit the formation of *N*-nitrozo compounds. However, *N*-nitrosation is catalyzed by monodihydroxyphenols and some polyhydroxyphenols. It is emphasized that this catalytic activity occurs when the concentration of the nitrosating agent is higher than the phenol concentration [47]. In a previous study, it was reported that the lack of increase in NDMA level in the ascorbate group at 15 g/kg black pepper level was probably due to the synergistic effect of both phenolic compounds in black pepper and ascorbate [16]. Studies have shown that the catalysis or inhibition of nitrosamine formation by phenolic compounds varies significantly depending on the structure of the compound and nitrite concentration, pH and reaction conditions [47,48].

#### 3.3.2. *N*-Nitrosodiethylamine (NDEA)

**Figure 2:**
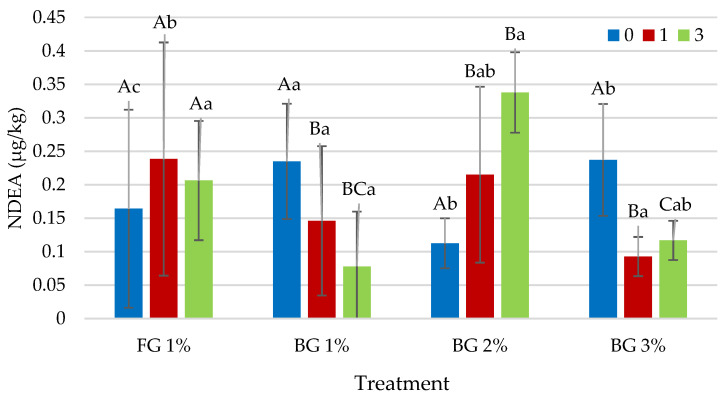
Effect of interaction of garlic treatment × cooking time on *N*-Nitrosodiethylamine (NDEA) content of heat-treated sucuk (A–C: Different capital letters indicate significant differences between groups with garlic for cooking time. a, b: Different small letters indicate significant differences between cooking time for garlic treatment).

It was determined that both garlic treatment and cooking time had a very significant (*p* < 0.01) effect on NDEA. In addition, the treatment × cooking time interaction was also effective on the NDEA content of the heat-treated sucuk groups at the *p* < 0.01 level (Table 3). The use of FG (1%) and BG (1%, 2% and 3%) had no significant effects on NDEA content in heat-treated sucuk when no additional cooking process (0 min) was applied (Figure 2). On the other hand, when cooking time was 1 min, the use of BG in heat-treated sucuk caused a significant decrease in NDEA content compared with FG. However, there were no significant differences between 1%, 2% and 3% levels of BG. In the 3 min cooking process, the group with 3% BG gave the lowest NDEA content (Figure 2). In the present study, the lower NDEA values of the samples containing BG were estimated to be related to both the residual nitrite level and the antioxidant activity of BG. Nitrosamine formation reactions are very complex and are highly influenced by factors such as residual nitrite, water activity, added nitrite level, nitrosation catalysts and inhibitors [15,49]. On the other hand, it was observed that FG and BG have different effects during cooking. In the presence of FG, there was an increase in NDEA content due to the increase in cooking time, while there were no significant effects of cooking time with the presence of 1% BG. While the difference between 0 min and 3 min was found to be significant at 2% BG, a significant difference was also found between 0 min and 1 min of cooking at 3% BG (Figure 2). Considering all these results, it can be said that BG is more effective in preventing NDEA formation during cooking. This result is probably due to the fact that BG contains different antioxidant substances [25,42].

#### 3.3.3. *N*-Nitrosopiperidine (NPIP)

NPIP is frequently detected in many processed meat products, including fermented sausages such as salami and cooked sausages, due to the use of black pepper [10]. Piperine (1-piperol-piperidine) and piperidine, a cyclic secondary amine found in black pepper, play an important role in NPIP formation [50]. In the present study, the average NPIP value was determined at the lowest level in the 1% FG group with 1.76 μg/kg. De Mey et al. [8] determined the NPIP content at 12.3 μg/kg in a product called pepper salami. In another study, it was reported that the NPIP content increased as the black pepper level in fermented sausage batter increased [9]. Sallan et al. [16] also reported that the use of black pepper in sausage increased the amount of NPIP, but high levels of black pepper decreased the NPIP content due to the antioxidant compounds found in this spice. In the present study, however, higher NPIP content was obtained in the 1% BG group compared with the 1% FG group, and NPIP content also increased in the presence of 2% or 3% BG levels (*p* < 0.01) (Table 3). In a study investigating the role of cadaverine and piperidine and the effects of pH, nitrite and ascorbate on NPIP formation in dry fermented sausage, it was reported that the role of ascorbate as a nitrosamine inhibitor could be seen only in the first step of manufacturing, and the level of ascorbate or nitrite at the beginning did not affect the NPIP in the end product [51]. In parallel with these results, in the present study, the fact that the NPIP content in heat-treated sucuk did not decrease with increasing BG levels is thought to be due to the inability of BG to show its inhibitory potential as an antioxidant or the expected effects in reducing the NPIP content with lower residual nitrite levels. On the contrary, substances that may contribute to the piperidine cycle required for NPIP formation may be present in the BG content.

In addition, as can be seen from Table 3, the NPIP content of the groups increased as the cooking time increased (*p* < 0.01) (Table 3). It has been determined in other studies that cooking intensity has a very important effect on NPIP content in sucuk and heat-treated sucuk [16,17]. In many other studies, it has been reported that the amount of nitrosamine increases with increasing cooking time [9,10,52,53,54,55]. NPIP has an important share among the determined nitrosamines. At the end of 3 min of cooking, the mean NPIP, NDMA and NDEA values were 5.61 μg/kg, 1.24 μg/kg and 0.86 μg/kg, respectively. As can be understood from these results (Table 3), the total nitrosamine content is below the limit value given for cured meat products in the United States even when cooked for 3 min [56].

**Table 3:** The overall effects of garlic treatment and cooking time on color value and the nitrosamine contents of heat-treated sucuk (mean ± sd).

Treatment (T)	Nitrosamines (μg/kg)		
	NDMA	NDEA	NPIP
FG (1%)	1.17 ± 0.40 a	1.01 ± 0.32 a	1.76 ± 1.36 c
BG (1%)	1.09 ± 0.38 ab	0.67 ± 0.09 b	3.05 ± 1.87 b
BG (2%)	1.01 ± 0.36 b	0.68 ± 0.11 b	4.36 ± 2.02 a
BG (3%)	0.62 ± 0.17 c	0.64 ± 0.06 b	4.21 ± 2.03 a
*Significance*	**	**	**
Cooking Time (CT)			
0 min	0.62 ± 0.19 c	0.61 ± 0.10 c	1.76 ± 1.05 c
1 min	1.05 ± 0.31 b	0.77 ± 0.21 b	2.66 ± 1.12 b
3 min	1.24 ± 0.38 a	0.86 ± 0.29 a	5.61 ± 1.65 a
*Significance*	**	**	**
*T × CT*	**	**	ns

FG: fresh garlic; BG: black garlic; ns: not significant; *p* > 0.05; **: *p* < 0.01; sd: standard deviation; a–c: means marked with different letters in the same column are statistically different (*p* < 0.05).

### 3.4. Heatmap

Cluster analysis based on the physicochemical parameters of heat-treated sucuk with garlic treatment was performed to show the general differences between the groups. To show the similarities between the heat-treated sucuk samples, a dendrogram was created using a heatmap clustering algorithm. The X and Y axes represent the physicochemical parameters and the garlic groups, respectively. The intensity of the yellow and blue colors were used for higher and lower correlation coefficients, respectively. For the representation of the heatmap, the average linkage was used for the cluster method and the Pearson correlation was chosen for the distance measurement.

**Figure 3:**
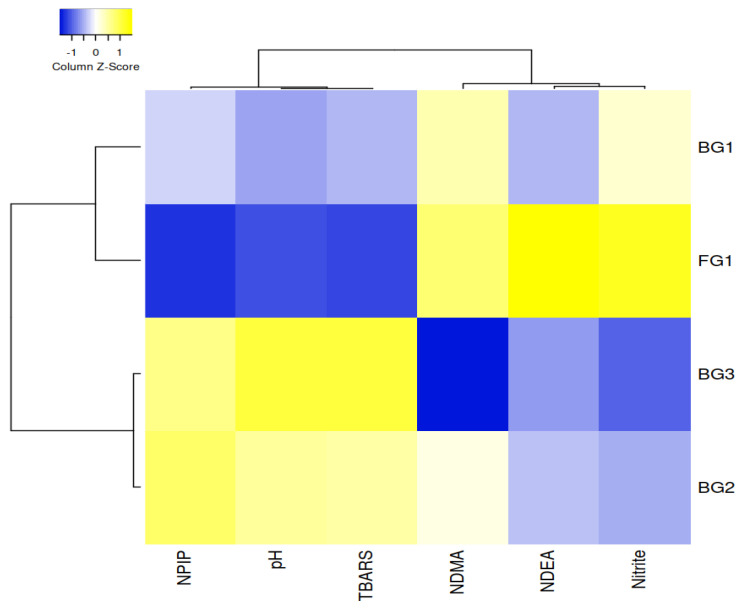
Cluster analysis of heatmap showing the relationship between physicochemical parameters and garlic treatments (The X and Y axes represent the physicochemical and garlic treatments, respectively (the intensity of the yellow and blue color represents from higher to lower correlation levels).

Figure 3 shows the two main clusters for groups with garlic (FG1, BG1, BG2 and BG3) and physicochemical parameters such as pH, TBARS, residual nitrite, NPIP, NDMA and NDEA. The first cluster included groups of BG2 and BG3, and the second cluster included groups of FG1 and BG1. Groups of BG2 and BG3 correlated negatively with nitrite and NDEA. Residual nitrite affected the nitrosamine levels [7,49]. As can be seen from Table 1, both BG2 and BG3 caused an important decrease in residual nitrite compared with FG1. On the other hand, BG2 and BG3 correlated positively with pH, TBARS and NPIP (Figure 3). The NPIP level also increased in these groups. Indeed, a positive correlation between NPIP and TBARS was also reported by Sallan [5].

**Figure 4:**
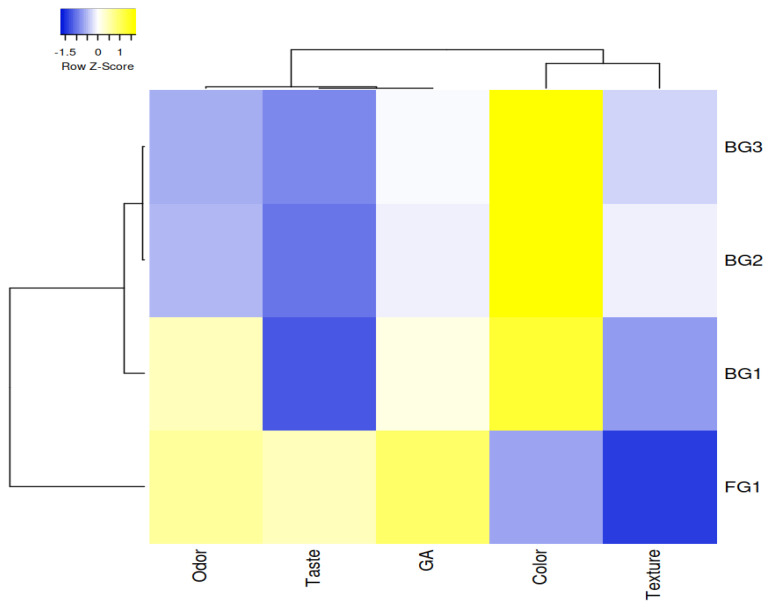
Cluster analysis of heatmap showing the relationship between sensory characteristics and garlic treatments (The X and Y axes represent the sensory characteristics and garlic treatments, respectively. (The intensity of the yellow and blue color represents from higher to lower correlation levels; GA: general acceptability).

Figure 4 illustrates the two main clusters for sensory analysis, in which the parameters are odor, taste, color, texture, general acceptability (GA) and groups with garlic (FG1, BG1, BG2 and BG3). FG1 participated in the first cluster, while BG1, BG2 and BG3 were taken as part of the second cluster, which included BG1 as a subgroup. FG1 positively correlated with odor, taste and general acceptability, while it negatively correlated with color and texture. BG1, BG2 and BG3 negatively correlated with taste, while they positively correlated with color. On the other hand, BG2 and BG3 negatively correlated with odor.

### 3.5. Principal Component Analysis (PCA)

**Figure 5:**
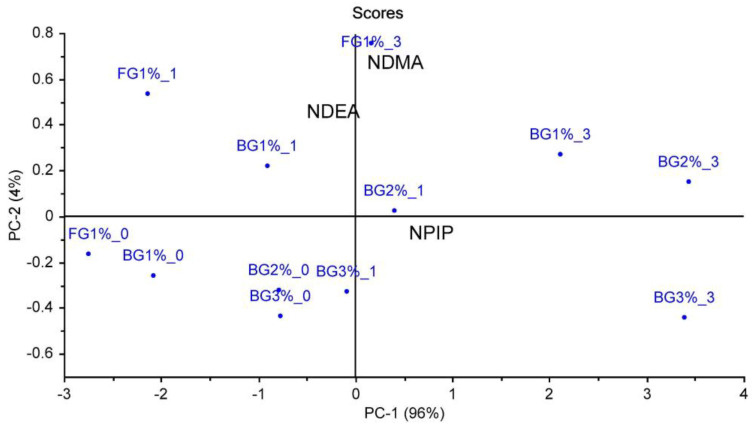
The result of principal component analysis of the relationships between factors (garlic treatment and cooking time) and nitrosamines.

PCA was used to evaluate the relationships between factors (garlic treatment and cooking time) and nitrosamines (Figure 5). The first PC (PC1) was enough to explain 96% of the variation. The PC2 explained 4% of the total variance. One hundred percent of the total variance was explained by the first two principal components. A positive correlation with NPIP and NDMA was determined after 3 min of cooking at both 1% FG and three different BG ratios, and these groups were on the positive part of PC1. The group containing 2% BG and cooked for 1 min (BG2%_1) was on the positive part of PC1 and positively correlated with NDMA and NPIP. In addition, a closer correlation of NPIP with groups containing BG was observed. As can be seen from Table 3, even %1 BG content caused a significant increase in NPIP content. In contrast, NDMA was more correlated with the FG group cooked for 3 min. It was also observed that there was a more positive correlation, especially with NPIP, after 1 min of cooking at the ratio of 2% BG. NDEA, together with uncooked and cooked for 1 min samples except for BG2%_1 min, was on the negative part of PC1, and NDEA was positively correlated with these groups. These results showed that cooking time was correlated more strongly with NDMA and NPIP. The relationship between cooking intensity and NDMA or NPIP was also determined in the previous studies on sucuk and heat-treated sucuk [13,16,17]. However, in the present study, no significant changes were detected in the presence of BG even after 3 min of cooking (Figure 1).

## 4. Conclusions

Although the use of BG at 2% and 3% caused a reduction in the number of lactic acid bacteria, sufficient acidification of the product was achieved. The use of BG decreased the number of micrococci and staphylococci. The TBARS value increased as the level of BG increased; however, no rancid taste was noted in the sensory evaluation. In terms of general acceptability, FG at 1% received the highest score, while the use of BG increased color scores. The use of BG decreased the residual nitrite, which was considered an important result in terms of nitrosamine formation. In the presence of FG1%, BG1% and BG2%, a cooking time of 1 or 3 min increased the NDMA content, whereas there were no cooking time-related changes in the presence of 3% BG. In terms of NDEA, 3% BG treatment gave the lowest value in 3 min of cooking. The use of BG caused an increase in NPIP, and particularly 2% BG cooked for 1 min correlated more strongly with NPIP. As a result, it was concluded that BG has a limited effect on quality characteristics of heat-treated sucuk and reduces the risk of nitrosamines by inhibiting the formation of NDMA and NDEA, which are group 2A carcinogenic compounds, during cooking. However, BG causes an increase in NPIP content.

## Figures and Tables

**Table 2:** The overall effect of garlic treatment on sensory characteristics of heat-treated sucuk (mean ± sd).

Treatment	Sensory Parameters				
	Color	Taste	Odor	Texture	General Acceptability
1% FG	7.52 ± 0.20 c	7.72 ± 0.10 a	7.75 ± 0.17 a	7.38 ± 0.16 a	7.82 ± 0.20 a
1% BG	7.68 ± 0.10 b	7.40 ± 0.20 b	7.58 ± 0.18 a	7.45 ± 0.05 a	7.55 ± 0.20 b
2% BG	7.85 ± 0.09 a	7.20 ± 0.05 b	7.33 ± 0.03 b	7.43 ± 0.10 a	7.43 ± 0.14 b
3% BG	7.98 ± 0.19 a	7.22 ± 0.21 b	7.30 ± 0.05 b	7.38 ± 0.08 a	7.47 ± 0.03 b
*Significance*	**	**	**	ns	*

FG: fresh garlic; BG: black garlic; ns: not significant; *: *p* < 0.05; **: *p* < 0.01; sd: standard deviation; a–c: means marked with different letters in the same column are statistically different (*p* < 0.05).

## Data Availability

The data presented in this study are available on request from the corresponding author.

## References

[1] Oral Z.F.Y., Kaban G. (2021). Effects of autochthonous strains on volatile compounds and quality properties of heat-treated sucuk. Food Biosci..

[2] Kaya M., Kaban M., Aran N. (2019). Fermente Et Ürünleri. Gıda Biyoteknolojisi.

[3] Alahakoon A.U., Jayasena D.D., Ramachandra S., Jo C. (2015). Alternatives to nitrite in processed meat: Up to date. Trends Food Sci. Technol..

[4] Sirini N., Munekata P.E.S., Lorenzo J.M., Stegmayer M.Á., Pateiro M., Pérez-Álvarez J.Á., Sepúlveda N., Sosa-Morales M.E., Teixeira A., Fernández-López J. (2022). Development of Healthier and Functional Dry Fermented Sausages: Present and Future. Foods.

[5] Sallan S. (2023). Influence of sheep tail fat and autochthonous starter culture on the formation of volatile nitrosamines in sucuk. Kafkas Univ. Vet. Fak. Derg..

[6] International Agency for Research on Cancer (IARC) (2022). Agents Classifed by the IARC Monographs, Volumes 1–128. https://monographs.iarc.who.int/list-of-classifications.

[7] Sallan S., Yılmaz Oral Z.F., Kaya M. (2023). A Review on the Role of Lactic Acid Bacteria in the Formation and Reduction of Volatile Nitrosamines in Fermented Sausages. Foods.

[8] De Mey E., De Klerck K., De Maere H., Dewulf L., Derdelinck G., Peeters M.-C., Fraeye I., Heyden Y.V., Paelinck H. (2014). The Occurrence Of N-Nitrosamines, Residual Nitrite And Biogenic Amines In Commercial Dry Fermented Sausages And Evaluation Of Their Occasional Relation. Meat Sci..

[9] Herrmann S.S., Granby K., Duedahl-Olesen L. (2015). Formation and mitigation of N-nitrosamines in nitrite preserved cooked sausages. Food Chem..

[10] Herrmann S.S., Duedahl-Olesen L., Granby K. (2015). Occurence of volatile and nonvolatile N-nitrosamines in processed meat products and role of heat treatment. Food Control.

[11] Özbay S., Şireli U.T. (2022). The effect of ascorbic acid, storage period and packaging material on the formation of volatile N-nitrosamine in sausages. J. Food Sci. Technol..

[12] Kaban G., Polat Z., Sallan S., Kaya M. (2022). The occurrence of volatile N-nitrosamines in heat-treated sucuk in relation to pH, a_w_ and residual nitrite. J. Food Sci. Technol..

[13] Kızılkaya M.F., Yılmaz Oral Z.F., Sallan S., Kaban G., Kaya M.V. (2023). Volatile nitrosamines in a dry fermented sausage: Occurrence and effect of cooking on their formation. J. Food Compos. Anal..

[14] Li L.L., Shao J.H., Zhu X.D., Zhou G.H., Xu X.L. (2013). Effect of plant polyphenols and ascorbic acid on lipid oxidation, residual nitrite and N-nitrosamines formation in dry-cured sausage. Int. J. Food Sci. Technol..

[15] Wang Y., Li F., Zhuang H., Chen X., Li L., Qiao W., Zhang J. (2015). Effects of plant polyphenols and α-tocopherol on lipid oxidation, residual nitrites, biogenic amines and n-nitrosamines formation during ripening and storage of dry-cured bacon. LWT.

[16] Sallan S., Kaban G., Kaya M. (2019). Nitrosamines in sucuk: Effects of black pepper, sodium ascorbate and cooking level. Food Chem..

[17] Sallan S., Kaban G., Sisik Oğras S., Çelik M., Kaya M. (2020). Nitrosamine formation in a semi-dry fermented sausage: Effects of nitrite, ascorbate and starter culture and role of cooking. Meat Sci..

[18] Sun F., Kong B., Chen Q., Han Q., Diao X. (2017). N-nitrosoamine inhibition and quality preservation of harbin dry sausages by inoculated with *Lactobacillus pentosus*, *Lactobacillus curvatus* and *Lactobacillus sake*. Food Control.

[19] Xiao Y.Q., Li P.J., Zhou Y., Ma F., Chen C.G. (2018). Effect of inoculating *Lactobacillus pentosus* R3 on N-nitrosamines and bacterial communities in dry fermented sausages. Food Control.

[20] Shao X., Zhu M., Zhang Z., Huang P., Xu B., Chen C., Li P. (2021). *N*-nitrosodimethylamine reduction by *Lactobacillus pentosus* R3 in fermented cooked sausages. Food Control.

[21] Shenoy N.R., Choughuley A.S.U. (1992). Inhibitory effect of diet related sulphydryl compounds on the formation of carcinogenic nitrosamines. Cancer Lett..

[22] Choi S.Y., Chung M.J., Lee S.-J., Shin J.H., Sung N.J. (2007). N-nitrosamine inhibition by strawberry, garlic, kale, and the effects of nitrite-scavenging and N-nitrosamine formation by functional compounds in strawberry and garlic. Food Control.

[23] Lanzotti V. (2006). The analysis of onion and garlic. J. Chromatogr. A.

[24] Kim J.-S., Kang O.-J., Gweon O.-C. (2013). Comparison of phenolic acids and flavonoids in black garlic at different thermal processing steps. J. Funct. Foods..

[25] Kimura S., Tung Y.-C., Pan M.-H., Su N.-W., Lai Y.-J., Cheng K.-C. (2017). Black garlic: A critical review of its production, bioactivity, and application. J. Food Drug Anal..

[26] Ríos-Ríos K.L., Montilla A., Olano A., Villamiel M. (2019). Physicochemical changes and sensorial properties during black garlic elaboration: A review. Trends Food Sci. Technol..

[27] Toldra F., Nip W.-K., Hui Y.H., Toldra F. (2017). Dry-fermented sausages: An overview. Handbook of Fermented Meat and Poultry.

[28] Incze K., Toldra F. (2017). European Products. Handbook of Fermented Meat and Poultry.

[29] Rust R.E., Toldra F. (2017). U.S. Products. Handbook of Fermented Meat and Poultry.

[30] Afzaal M., Saeed F., Rasheed R., Hussain M., Aamir M., Hussain S., Mohamed A.A., Alamri M.S., Anjum F.M. (2021). Nutritional, biological, and therapeutic properties of black garlic: A critical review. Int. J. Food Prop..

[31] Armutçu Ü., Hazar F.Y., Yılmaz Oral Z.F., Kaban G., Kaya M. (2020). Effects of different internal temperature applications on quality properties of heat-treated sucuk during production. J. Food Process. Preserv..

[32] Kaban G., Yılmaz Oral Z.F., Kaya M., Lorenzo J.M., Dominguez R., Pateiro M., Munekata P.E.S. (2022). Sucuk. Production of Traditional Mediterranean Meat Products.

[33] Kaya M., Güllüce M., Kaban G., Çınar K., Karadayı M., Bozoglu C., Sayın B., Alaylar B. (2017). The usage possibilities of lactic acid bacteria and coagulase negative staphylococcus strains isolated from traditional sucuk as starter culture. TAGEM-13/ARGE/7 (Final Report).

[34] Kaban G., Kaya M. (2008). Identification of lactic acid bacteria and Gram-positive catalase-positive cocci isolated from naturally fermented sausage (sucuk). J. Food Sci..

[35] Kaban G., Sallan S., Çinar Topçu K., Sayın Börekçi B., Kaya M. (2022). Assessment of technological attributes of autochthonous starter cultures in Turkish dry fermented sausage (sucuk). Int. J. Food Sci. Technol..

[36] Lemon D.W. (1975). An Improved TBA Test for Rancidity New Series Circular.

[37] NKML (Nordic Committee of Food Analysis) (2000). Nitrite and Nitrate in Foodstuffs by Ion Chromatography.

[38] Babicki S., Arndt D., Marcu A., Liang Y., Grant J.R., Maciejewski A., Wishart D.S. (2016). Heatmapper: Web-enabled heat mapping for all. Nucleic Acids Res..

[39] Kaban G., Bayraktar F., Jaberi R., Fettahoğlu K., Kaya M. (2022). Effects of NaCl substitution with KCl on quality properties of heat-treated sucuk during the production stages. J. Food Nutr. Res..

[40] Kaban G., Bayrak D. (2015). The effects of using Turkey meat on qualitative properties of heat-treated sucuk. Czech J. Food Sci..

[41] Lishianawati T.U., Yusiati L.M., Jamhari (2022). Antioxidant effects of black garlic powder on spent duck meat nugget quality during storage. Food Sci. Technol..

[42] Qiu Z., Zheng Z., Zhang B., Sun-Waterhouse D.-S., Qiao X. (2020). Formation, nutritional value, and enhancement of characteristic components in black garlic: A review for maximizing the goodness to humans. Compr. Rev. Food Sci. Food Saf..

[43] Ahmed T., Wang C.-K. (2021). Black Garlic and Its Bioactive Compounds on Human Health Diseases: A Review. Molecules.

[44] Shin D.-M., Hwang K.-E., Lee C.-W., Kim T.-K., Park Y.-S., Han S.G. (2017). Effect of Swiss Chard (Beta vulgaris var. cicla) as Nitrite Replacement on Color Stability and Shelf-Life of Cooked Pork Patties during Refrigerated Storage. Korean J. Food Sci. An..

[45] Choi S., Cha H.S., Lee Y.S. (2014). Physicochemical and antioxidant properties of black garlic. Molecules.

[46] Belitz H.D., Grosch W., Schieberle P. (2001). Lehrburch der Lebensmittelchemie.

[47] Gonzalez-Mancebo S., Garcia-Santos M.P., Hernandez-Benito J., Calle E., Casado J. (1999). Nitrosation of phenolic compounds: Inhibition and enhancement. J. Agric Food Chem..

[48] Shenoy N.R., Choughuley A.S.U. (1989). Effect of certain phenolics on nitrosamine formation. J. Agric. Food Chem..

[49] De Mey E., De Maere H., Paelinck H., Fraeye I. (2017). Volatile N-nitrosamines in meat products: Potential precursors, influence of processing, and mitigation strategies. Crit. Rev. Food Sci. Nutr..

[50] De Mey E., De Maere H., Dewulf L., Paelinck H., Sajewicz M., Fraeye I., Kowolska T. (2014). Assessment of the *N*-nitrosopiperidine formation risk from piperine and piperidine contained in spices used as meat product additives. Eur. Food Res. Technol..

[51] De Mey E., De Maere H., Goemaere O., Steen L., Peeters M.-C., Derdelinckx G., Paelinck H., Fraeye I. (2014). Evaluation of n-nitrosopiperidine formation from biogenic amines during the production of dry fermented sausages. Food Bioproc. Tech..

[52] Sen N.P., Donaldson B.A., Seaman S., Iyengar J.R., Miles W.F. (1976). Inhibition of nitrosamine formation in fried bacon by propyl gallate and L-ascorbyl palmitate. J. Agric. Food Chem..

[53] Sen N.P., Seaman S., Miles W.F. (1979). Volatile nitrosamines in various cured meat products: Effect of cooking and recent trends. J. Agric. Food Chem..

[54] Rywotycki R. (2007). The effect of baking of various kinds of raw meat from different animal species and meat with functional additives on nitrosamine contamination level. Food Chem..

[55] Yurchenko S., Mölder U. (2007). The occurence of volatile N-nitrosamines in Eastonian meat products. Food Chem..

[56] Crews C. (2010). Determination of nitrosamines in food. Qual. Assur. Saf. Crops Foods..

